# Anisogamy and the Darwin–Bateman paradigm

**DOI:** 10.1093/evlett/qrae044

**Published:** 2024-08-17

**Authors:** Tim Janicke

**Affiliations:** CEFE, University of Montpellier, CNRS, EPHE, IRD, Montpellier, France

**Keywords:** Bateman gradient, Bateman’s principle, gamete size, sexual selection

## Abstract

The Darwin–Bateman paradigm advanced as the central concept to explain the evolutionary origin of sex differences. However, debates regarding its theoretical underpinnings persist, particularly with respect to the role of anisogamy in sexual selection. The theoretical work presented by Lehtonen and Parker suggests that the initial split in gamete production rate drives sex differences in sexual selection but that any further variation in the degree of anisogamy is not expected to alter the strength of sexual selection in males and females. Here, I discuss the historical background of a recently emerged controversy and present empirical data that corroborate the theoretical predictions. Lehtonen and Parker’s contribution refines our understanding of the Darwin–Bateman paradigm by providing a broad theory for large-scale patterns of sex differences that can be observed in nature. Further understanding of how demographic and environmental factors influence sexual selection is essential to predict the vast diversity of sex differences across the tree of life, beyond the primordial impact of anisogamy.

## Introduction

The Darwin–Bateman paradigm has become a cornerstone in evolutionary biology by providing a unifying framework to explain the tremendous diversity of sex differences in morphologies, behaviors and life-history traits. Yet, Darwin’s and Bateman’s original ideas have been subject to recurrent debates centering primarily on the conjecture that sex differences have a shared evolutionary origin (Schärer et al., 2012). This controversy persists until today, partly because some of the core concepts rely on verbal arguments rather than explicit theoretical foundations. The study by Lehtonen and Parker (2024) (hereafter L&P) is a rare attempt to explore the underlying theoretical basis for the role of anisogamy in the evolution of sex differences, enabling us to better contextualize emerging criticism of the Darwin–Bateman paradigm.

One recent criticism of the paradigm comes from a comparative study by Mokos et al. (2021), which did not find evidence for a relationship between the degree of anisogamy and standardized metrics of sexual selection across a broad range of exclusively anisogamous species, spanning insects, echinoderms, fish, amphibians, reptiles, and mammals. Under the premise that the Darwin–Bateman paradigm implies an effect of anisogamy on the sex difference in sexual selection, the authors claimed that their finding questions the validity of the initial pathway of the paradigm and, as a consequence, the study became a reference for challenging the foundations of sexual selection theory (Andersson et al., 2023). Mokos et al.’s (2021) work expanded on a previous meta-analysis in which my colleagues and I examined whether sexual selection typically varies between the sexes (Janicke et al., 2016). Despite significant among-species diversity in the estimated sexual selection proxies, we found support for Darwin’s and Bateman’s claim that sexual selection is typically stronger in the male sex. Given that the biological sex is defined by the difference in gamete size, we argued that the detected sex difference in sexual selection must be rooted in anisogamy and claimed that our findings, therefore, corroborate the initial pathway of the Bateman–Darwin paradigm. Consequently, the conclusions derived from the two empirical studies, which are based on nearly the same set of sampled species, appear contradictory at first glance. Fortunately, L&P’s contribution now clarifies these apparent discrepancies convincingly.

## A brief history of the Darwin–Bateman paradigm

Before exploring the implications of L&P’s theoretical work, it might be helpful to outline the history of the Darwin–Bateman paradigm because I suspect that part of the mentioned controversy arose from misinterpreting the verbal arguments underlying the questioned concept. Dewsbury (2005) was inspired by the critical account of sexual selection theory by Hrdy (1986) when he coined the term “Darwin–Bateman paradigm” as “*the aggregate of the ideas that a.) male variance of reproductive success exceeds that of females, b.) males have more to gain from multiple matings, and c.) males are generally ardent and females coy*.” Hrdy’s (1986) thoughtful critique focused mainly on the main conclusions that Bateman (1948) draw from his classic experiment with fruit flies, which she originally conflated with the label “Bateman paradigm” and which Dewsbury (2005) renamed to “Darwin–Bateman paradigm” to acknowledge Darwin’s contribution. Importantly, Dewsbury’s original phrasing lacked any reference on the role of anisogamy, which prompted Parker and Birkhead (2013) to extend the Darwin–Bateman paradigm with the additional notion that the postulated sex differences are ultimately rooted in anisogamy. This extension appears more than justified because one of Bateman’s major contributions was to nominate sex-specific gamete production as the primordial driver for sex differences in sexual selection. Bateman (1948) argued “*The primary cause of intra-masculine selection would thus be seen to be that females produce much fewer gametes than males. Consequently there is competition between male gametes for the fertilization of the female gametes.*” Further he wrote “*The primary feature of sexual reproduction is to be sure the fusion of gametes irrespective of their relative size, but the specialization into large immobile gametes and small mobile gametes produced in great excess (the primary sex difference), was a very early evolutionary step. One would therefore expect to find in all but a few very primitive organisms, and those in which monogamy combined with a sex ratio of unity eliminated all intra-sexual selection, that males would show greater intra-sexual selection than females*.” Bateman may have adopted an overly generalized perspective when claiming that all anisogamous species with a polygamous mating system are expected to exhibit stronger sexual selection in males. Yet, the core idea is both simple and ingenious: males produce more gametes than females, leading to stronger competition among males to fertilize female gametes. Thus, the numerical imbalance of male and female gametes—the key characteristic of anisogamous species—makes males generally more prone to compete for females.

To recapitulate, the original verbalization of the “Darwin–Bateman paradigm” did not address the role of anisogamy for sex differences despite the fact that Bateman alluded very precisely that stronger sexual selection in males is rooted in sex differences in gamete production. Therefore, Parker and Birkhead’s (2013) proposition to incorporate the impact of anisogamy into the Darwin–Bateman paradigm was an adequate and necessary extension of the concept, but the postulated role concerns the primordial effect of anisogamy on the expected overall sex difference in sexual selection. Bateman did not predict that inter-specific variation in the degree of anisogamy affects the strength of sexual selection. In fact, he considered that sexual selection is stronger in males in nearly all anisogamous organisms except for monogamous species with an even sex ratio and species that he considered “very primitive”, presumably referring to isogamous species that do not show the described specialization of gametes.

## Controversy? What controversy?

In an earlier meta-analysis, we followed Bateman’s rationale to test whether sex—defined by the difference in gamete size within each sampled species—predicts the strength of sexual selection (Janicke et al., 2016). We found that males show typically signatures of stronger sexual selection than females, which we interpreted as support for the idea that gamete size is key to understanding the diversity in sexual selection as postulated by the Darwin–Bateman paradigm. Admittedly, the obtained evidence is limited because it is only correlational and ideally one would like to study sex differences in sexual selection of replicated lineages with independent evolutionary origins of anisogamy. In their more recent comparative study, Mokos et al. (2021) aimed at testing whether the magnitude of the sex difference in gamete size is associated with sex differences in the strength of sexual selection. The authors stated that this relationship is a “crucial assumption” of the Darwin–Bateman paradigm, and the observed absence of an evolutionary link between the degree of anisogamy and sex differences in sexual selection would challenge the initial step of the paradigm. As outlined above, I believe that the hypothesis tested by Mokos et al. (2021) has never been an integral part of the Darwin–Bateman paradigm, and therefore, their findings can neither support nor challenge its validity. However, this does not mean that the tested hypothesis by Mokos et al. (2021) does not address an interesting question. Understanding the diversity of sexual selection across the animal tree of life has always been a prominent theme in evolutionary biology, and Mokos et al.’s (2021) work certainly added a new piece to the puzzle.

## Lehtonen and Parker’s broad theoretical predictions

**Figure 1.**
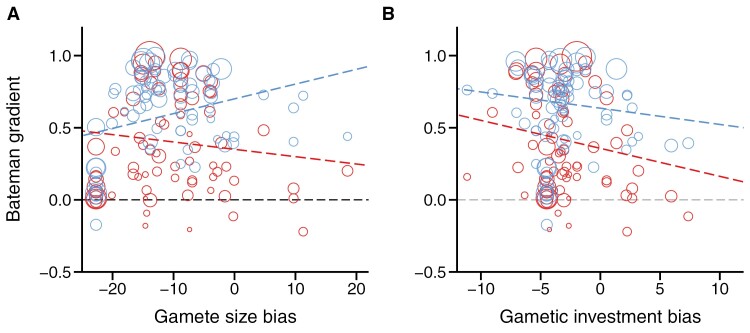
Degree of anisogamy and Bateman gradients across animals. Scatter plots show how Bateman gradients of males (blue) and females (red) relate to estimates of anisogamy in terms of gamete size bias (A) and gametic investment bias (B). Higher values indicate increased size of or investment in male gametes and are therefore representative of more isogamous species. Batmen gradients are shown as Pearson’s correlation coefficients (*r*) of the relationship between reproductive success and mating success. The radius of circles corresponds to the precision of the shown estimates of Bateman gradients (i.e., the inverse of the sampling variance in *r*). Phylogenetically informed meta-analyses suggest that estimates of gamete size ratio and gametic investment ratio are not related to male and female Bateman gradients, i.e., all shown slopes (dashed lines) are not significantly different from zero.

Regardless of its connection to the Darwin–Bateman paradigm, anisogamy may, in principle, influence the strength of sexual selection at various scales, but explicit mathematical modeling on this issue is surprisingly rare. L&P’s contribution is now filling an important gap by applying simple mechanistic evolutionary models to explore the causal relationship between gamete size dimorphism and pre-copulatory sexual selection at (i) the transition from isogamy to anisogamy and (ii) after strong anisogamy already evolved. First, building on earlier work (Lehtonen, 2022), L&P demonstrate that divergence in gamete number production generates divergent pre-copulatory sexual selection in terms of higher benefit of having an additional mating partner (i.e., a steeper “Bateman gradient”) in the sex with the higher gamete production rate. The argumentation closely follows that of Bateman: once gametes of one sex outnumber those of the other, only one sex can fertilize all gametes of the opposite sex in a single mating, whereas the other sex will face a surplus of gametes. This surplus of gametes imposes selection for the acquisition of additional mating partners and thus promotes the allocation of resources into pre-copulatory sexual competition in the sex with the higher gamete production rate. Assuming that gamete production trades-off with gamete size, the sex with surplus of gametes produces the smaller gametes, which is by definition the male. At a high fertilization success, the sex with the lower gamete production rate, which is, by definition, the female, will benefit from an additional mating only at very low levels of anisogamy. Importantly, with increasing sex difference in gamete production rate, the system quickly saturates such that the strength of selection does not diverge significantly beyond an early split. This saturation effect relies on the observation that the proportion of unfertilized male gametes asymptotes to one very quickly before reaching typical metazoan anisogamy ratios. Thus, the model predicts that anisogamy causes a binary split with stronger pre-copulatory sexual selection operating in males but that the correlation between the degree of anisogamy and sexual selection vanishes rapidly and becomes undetectable under typical gamete number ratios (see Figure 1 in L&P).

Investment in reproduction is often assumed to be fixed such that gametic expenditure is expected to trade-off with the pool of resources allocated toward pre-copulatory competitiveness (Parker, 2014; Simmons et al., 2017). In a second line of reasoning, L&P argue that the degree of anisogamy remains decoupled from the strength of pre-copulatory sexual selection under such a trade-off for two reasons. Firstly, even if males invest less into sperm, sperm competition, and/or sperm limitation are likely to maintain a surplus of male gametes. Both selective forces could drive increased gamete expenditure and, therefore, potentially relax pre-copulatory sexual selection. However, earlier work suggests that changes in the degree of anisogamy within the typical range will have a negligible effect on male gametic investment (Lehtonen et al., 2016). Secondly, L&P argue that the trade-off between gamete size and number implies that varying gamete size will change gamete number but not overall gametic expenditure. Yet, it is only overall gametic expenditure that dictates the amount of resources that can be reallocated toward mate acquisition (discussed by L&P in terms of “time out” and “time in,” respectively) such that changing sperm or ovum size does not necessarily affect the intensity of pre-copulatory sexual selection. Specifically, male gametic investment is predicted to increase with higher levels of sperm competition and sperm limitation, but the authors demonstrate that optimal sperm size can evolve independently from the level of sperm competition and is therefore unrelated to the expected investment in pre-copulatory sexual selection (i.e., “time in”).

The work of L&P is one of a few recent theoretical models that shed light on the primordial role of anisogamy in the evolution of sex roles. Building on Maynard–Smith’s game-theoretic framework (Maynard Smith, 1977), Iyer et al. (2020) found that smaller gametic investment by males can select for either symmetric parental investment or male-biased care. By contrast, assuming that parental investment coevolves with investment in sexually selected traits, Fromhage and Jennions (2016) found support for Trivers’ verbal arguments (Trivers, 1972) that an initial sex difference in parental investment promotes further sex-role divergence with the sex investing less to become more competitive. Irrespective the implications for parental care, Siljestam and Martinossi-Allibert (2024) took the perspective of externally fertilizing organisms facing gamete limitation—a typical scenario for sessile marine broadcast spawners, which might represent the ancestral stage at the transition from isogamy to anisogamy. Their study suggests that anisogamy does not always promote male-biased sexual competition because gamete limitation imposed by low densities might favor the evolution of competition traits in both sexes (Siljestam & Martinossi-Allibert, 2024). This illustrates that L&P’s work does not necessarily provide an exhaustive framework to explain the entire complexity of sexual systems in nature (see also Henshaw et al. (2022)) but rather offers valuable broad predictions for large-scale patterns on the evolutionary link between anisogamy and sex-specific sexual selection.

The predictions of L&P’s mathematical models match the available empirical data that have been obtained from comparative approaches: males and females differ in the strength of pre-copulatory sexual selection in terms of typically higher male benefits of having more than one mating partner (Janicke et al., 2016). However, among species with high levels of anisogamy (i.e., the vast majority of all metazoans), there is no relationship between the gamete-size ratio and pre-copulatory sexual selection (Mokos et al., 2021). Until now, the level of anisogamy was only found to correlate with sex-specific sexual selection across species with unusually low levels of anisogamy, such as fruit flies with so-called “giant sperm” (Bjork & Pitnick, 2006).

The findings by Mokos et al. (2021) do not question the Darwin–Bateman paradigm because they address a hypothesis that is not part of that concept and they are in line with the set of predictions generated by L&P. Yet, Mokos et al. (2021) do not provide a direct test of the proposed theory because they explored how the degree of anisogamy is related to an effect size quantifying the sex difference of the Bateman gradient whereas L&P explored how anisogamy relates to sex-specific male and female Bateman gradients. Higher values of the sex difference in the Bateman gradient can result not only from a steeper relationship between mating success and reproductive success in males compared to females but also from a shallow Bateman gradient in males and a negative relationship in females (Devost & Turgeon, 2016). Similarly, small effect sizes might indicate a shallow Bateman gradient in males, but they can also arise from steep Bateman gradients in both sexes (Winkler et al., 2023). Therefore, the imperfect correlation between male or female Bateman gradients and the effect size used in Mokos et al. (2021) might have obscured the tested relationship between sexual selection and anisogamy. In an attempt to provide a more direct empirical test of L&Ps predictions, I adopted Mokos et al.’s (2021) approach and tested how their compiled estimates of the gamete size bias and gametic investment bias are correlated with male and female Bateman gradients rather than their difference (for details, see Supplementary material). In the context of the Darwin–Bateman paradigm, I argue that estimates of gamete size bias (computed as log([sperm mass/male mass]/[egg mass/female mass])) appear to provide more informative proxies for degree of anisogamy than estimates of gametic investment bias (computed as log([testis mass/male mass]/([egg mass × clutch size]/female mass])). However, for the sake of completeness, I present analyses of the two metrics tested in Mokos et al. (2021). In total, I compiled 76 estimates for male and female Bateman gradients of the 48 species tested in Mokos et al. (2021). Based on this relatively small subset of species covered by Janicke et al. (2016), I could confirm the earlier finding that the Bateman gradient is typically stronger in males than females (males: *r* ± *SE* = 0.657 ± 0.205; females: *r* ± *SE* = 0.378 ± 0.231; Omnibus test for sex difference: *QM* = 98.01, *N* = 152, *p* < 0.001). Importantly, gamete size bias was not significantly associated with Bateman gradients in males (estimate ± *SE* = 0.010 ± 0.008, *N* = 76, *z*-value = 1.253, *p* = 0.210) or females (estimate ± *SE* = −0.005 ± 0.010, *N* = 76, *z*-value = −0.518, *p* = 0.605) (Figure 1A). Similarly, I did not detect a statistically significant relationship between gametic investment bias and Bateman gradients in males (estimate ± *SE* = −0.011 ± 0.014, *N* = 76, *z*-value = −0.805, *p* = 0.421) and females (estimate ± *SE* = −0.019 ± 0.019, *N* = 76, *z*-value = −1.050, *p* = 0.294) (Figure 1B). These findings are in line with results by Mokos et al. (2021) and support L&Ps predictions that the sex difference in gamete size is unrelated to pre-copulatory sexual selection across species showing typical levels of anisogamy.

The work by L&P provides the much-needed theoretical framework for predicting the effect of anisogamy on sexual selection. Yet, the authors remind us repeatedly that their models are simplistic and based on basic assumptions to infer broad predictions. Thus, readers should not be surprised that they do not capture the entire diversity of sex roles in sexually reproducing organisms. Obvious deviations from the predicted trajectories are so called sex-role reversed species in which females are subject to stronger sexual selection than males despite typical anisogamy ratios (Fritzsche et al., 2021) or the observation that the female Bateman gradient is typically positive across species with highly male-biased gamete size ratios (Fromonteil et al., 2023). In this context, the authors of all three discussed studies concur that more accurate predictions of the vast diversity of sexual systems hinge on a deeper understanding of how demographic and environmental factors contribute to sex differences in sexual selection.

## Supplementary material

Supplementary material is available online at *Evolution Letters*.

qrae044_suppl_Supplementary_Material

## Data Availability

The data underlying this article are available in the Dryad Digital Repository at https://doi.org/10.5061/dryad.z8w9ghxnr.
