# Supplementary material for: Anisogamy and the Darwin–Bateman paradigm
Source: Evol Lett. 2024 Aug 17;8(6):756–60. doi: 10.1093/evlett/qrae044 (PMC11637679; doi:10.1093/evlett/qrae044)
Supplement: qrae044_suppl_Supplementary_Material [file qrae044_suppl_supplementary_material.docx]

Supplementary Information for

**Anisogamy and the Darwin-Bateman Paradigm**

Tim Janicke^1*^

^1^ CEFE, Univ Montpellier, CNRS, EPHE, IRD, Montpellier, France.

*Corresponding author: tim.janicke@cefe.cnrs.fr

# SI 1. Phylogenetically informed meta-regression on the relationship between proxies of anisogamy and Bateman gradients

The meta-analysis testing the relationship between Bateman gradients and proxies of anisogamy rely entirely on data collected in previous studies. In particular, estimates of male and female Bateman gradients (i.e., Pearson correlation coefficients of the relationship between mating success and reproductive success) were obtained from Janicke et al. (2016). Details of the search protocol, inclusion criteria and PRISMA diagram can be found therein. Proxies of anisogamy are based on an earlier study by Mokos et al. (2021) and were kindly provided by the authors upon request. Specifically, anisogamy was estimated as the gamete size bias (GSB) index defined as

$$\text{GSB=log}\left( \frac{\left[ \frac{\text{male gamete mass}}{\text{male mass}} \right]}{\left[ \frac{\text{female gamete mass}}{\text{female mass}} \right]} \right)$$

and the gametic investment bias (GIB) computes as

$$\text{GIB=log}\left( \frac{\left[ \frac{\text{testis mass}}{\text{male mass}} \right]}{\left[ \frac{\text{female gamete mass×clutch size}}{\text{female mass}} \right]} \right)$$

Note that higher values of GSB and GIB indicate a greater male bias in gamete size and gametic investment, respectively, which is characteristic of more isogamous species. On the assumption of a trade-off between gamete size and number, these estimates are expected to be negatively correlated with the gamete-size ratio studied in Lehtonen and Parker (2024). Details of data extraction and data imputation to obtain estimates of GSB and GIB can be found in the Supplementary Material of Mokos et al. (2021). To account for phylogenetic non-independence, I used a dated phylogenetic tree of the sampled species that has been generated for Janicke et al. (2016) and later updated in Fromonteil et al. (2023). The used phylogenetic information is based on divergence times retrieved from the TimeTree database (Kumar et al., 2022).

In total, I obtained estimates of GSB and GIB from 48 species for which 76 estimates of Bateman gradients were available for males and females. Statistical analysis was carried out using a restricted maximum likelihood approach implemented in the *metafor* R package (Viechtbauer, 2010). First, I tested whether Bateman gradients differed between sexes by running multilevel linear mixed effects models with the rrma.mv function in which I defined the Pearson correlation coefficient as the response variable weighted by the inverse of its variance and sex as the only fixed effect together with observation identifier, study identifier and the correlation matrix of phylogenetic distances as random terms. Second, I ran separate models for each sex in which I tested the relationship between a given anisogamy proxy and Bateman gradients. Specifically, I defined the Pearson correlation coefficient of the Bateman gradient as the response variable and GSB or GIB as the fixed predictor variable together with observation identifier, study identifier and the correlation matrix of phylogenetic distances as random terms.

# References

Fromonteil S, Marie-Orleach L, Winkler L, Janicke T, 2023. Sexual selection in females and the evolution of polyandry. PLoS Biol 21:e3001916.

Janicke T, Häderer IK, Lajeunesse MJ, Anthes N, 2016. Darwinian sex roles confirmed across the animal kingdom. Sci Adv 2:e1500983.

Kumar S, Suleski M, Craig JM, Kasprowicz AE, Sanderford M, Li M, Stecher G, Hedges SB, 2022. TimeTree 5: an expanded resource for species divergence times. Mol Biol Evol 39:msac174.

Lehtonen J, Parker GA, 2024. The correlation between anisogamy and sexual selection intensity – the broad theoretical predictions. Evol Lett:qrae029. doi: <https://doi.org/10.1093/evlett/qrae029>.

Mokos J, Scheuring I, Liker A, Freckleton RP, Székely T, 2021. Degree of anisogamy is unrelated to the intensity of sexual selection. Sci Rep 11:19424.

Viechtbauer W, 2010. Conducting Meta-Analyses in R with the metafor Package. Journal of Statistical Software 36:1-48.
